# Retrospective Study of the Correlation Between Pathological Tumor Size and Survival After Curative Resection of T3 Pancreatic Adenocarcinoma: Proposal for Reclassification of the Tumor Extending Beyond the Pancreas Based on Tumor Size

**DOI:** 10.1007/s00268-017-4077-5

**Published:** 2017-06-15

**Authors:** Masanao Kurata, Goro Honda, Yoshiaki Murakami, Kenichiro Uemura, Sohei Satoi, Fuyuhiko Motoi, Masayuki Sho, Ippei Matsumoto, Manabu Kawai, Hiroaki Yanagimoto, Takumi Fukumoto, Minako Nagai, Masahiko Gosho, Michiaki Unno, Hiroki Yamaue, for the Multicenter Study Group of Pancreatobiliary Surgery (MSG-PBS)

**Affiliations:** 1 Department of Gastrointestinal and Hepato-biliary-Pancreatic Surgery, University of Tsukuba, 1-1-1 Tennodai, Tsukuba, 305-8575 Japan; 2 Department of Surgery, Tokyo Metropolitan Cancer and Infectious Diseases Center Komagome Hospital, Tokyo, Japan; 3 Department of Surgery, Institute of Biomedical and Health Sciences, Hiroshima University, Hiroshima, Japan; 4 Department of Surgery, Kansai Medical University, Osaka, Japan; 5 Division of Gastroenterological Surgery, Department of Surgery, Tohoku University, Sendai, Japan; 6 Department of Surgery, Nara Medical University, Nara, Japan; 7 Division of Hepato-Biliary-Pancreatic Surgery, Department of Surgery, Kobe University Graduate School of Medicine, Kobe, Japan; 8 Second Department of Surgery, Wakayama Medical University, Wakayama, Japan; 9 Department of Clinical Trial and Clinical Epidemiology, University of Tsukuba, Tsukuba, Japan

## Abstract

**Background:**

Even though most patients who undergo resection of pancreatic adenocarcinoma have T3 disease with extra-pancreatic tumor extension, T3 disease is not currently classified by tumor size. The aim of this study was to modify the current TNM classification of pancreatic adenocarcinoma to reflect the influence of tumor size.

**Methods:**

A total of 847 consecutive pancreatectomy patients were recruited from multiple centers. Optimum tumor size cutoff values were calculated by receiver operating characteristics analysis for tumors limited to the pancreas (T1/2) and for T3 tumors. In our modified TNM classification, stage II was divided into stages IIA (T3aN0M0), IIB (T3bN0M0), and IIC (T1-3bN1M0) using tumor size cutoff values. The usefulness of the new classification was compared with that of the current classification using Akaike’s information criterion (AIC).

**Results:**

The optimum tumor size cutoff value distinguishing T1 and T2 was 2 cm, while T3 was divided into T3a and T3b at a tumor size of 3 cm. The median survival time of the stages IIA, IIB, and IIC were 44.7, 27.6, and 20.3 months, respectively. There were significant differences of survival between stages IIA and IIB (*P* = 0.02) and between stages IIB and IIC (*P* = 0.03). The new classification showed better performance compared with the current classification based on the AIC value.

**Conclusions:**

This proposed new TNM classification reflects the influence of tumor size in patients with extra-pancreatic tumor extension (T3 disease), and the classification is useful for predicting mortality.

## Introduction

Pancreatic adenocarcinoma is the most lethal common cancer and the eighth leading cause of cancer-related death in men and the ninth leading cause of death in women throughout the world [1]. It has a very poor prognosis, with a 1-year survival rate of 25% and 5-year survival rate of 5%. Surgical resection is the only potential curative therapy for the tumor; however, only 15–20% of patients are considered to be candidates for resection because of an advanced stage at the time of diagnosis [2]. Many previous studies on the outcomes after curative resection of pancreatic adenocarcinoma have revealed that tumor size is an independent prognostic factor, along with lymph node metastasis and the surgical margin after curative resection of the tumor [3–13]. In the current TNM classification of the International Union Against Cancer (UICC) and the American Joint Committee on Cancer (AJCC), tumors limited to the pancreas are divided into T1 and T2 by tumor size (2 cm). On the other hand, there is no classification for tumor sizes extending beyond the pancreas (T3 and T4), despite the fact that most pancreatic adenocarcinomas that are resected are pathologically staged to T3 after surgery [14, 15].

In the present study, we reviewed all data from patients with pancreatic adenocarcinoma who had undergone pancreatectomy with curative intent to calculate tumor size cutoff values (Tco values) for tumors extending beyond the pancreas. We developed a modified TNM classification that incorporated the Tco values for T3 disease and statistically evaluated the usefulness of our proposed new TNM classification.

## Patients and methods

**Fig. 1:**
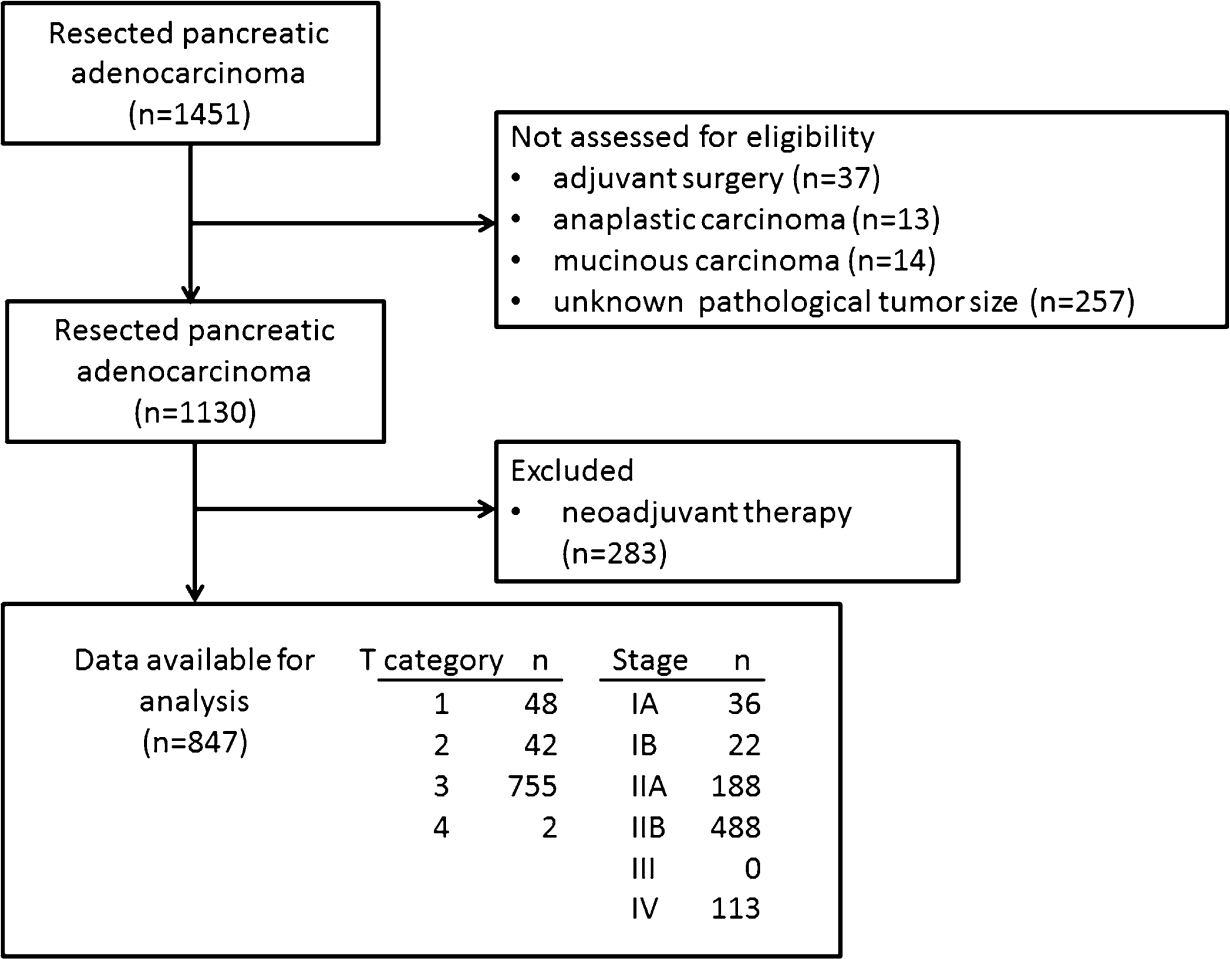
Flow diagram of the present study

Data were collected for a total of 1451 consecutive patients with pancreatic adenocarcinomas who underwent pancreatectomy with curative intent between 2001 and 2012 at seven high-volume surgical institutions in Japan (Tokyo Metropolitan Komagome Hospital, Hiroshima University Hospital, Nara Medical University Hospital, Tohoku University Hospital, Kansai Medical University Hospital, Kobe University Hospital, and Wakayama Medical University Hospital). All patients underwent R0 or R1 pancreatectomy and had a confirmed pathological diagnosis. Of the 1451 patients, we excluded 37 patients with initially unresectable tumors who received pancreatectomy after chemotherapy or chemoradiotherapy, 14 patients with mucinous carcinoma, 13 patients with anaplastic carcinoma, and 257 patients whose pathological tumor size was not recorded. In addition, 283 patients with resectable or borderline resectable tumors who received neoadjuvant therapy were excluded because it was considered difficult to accurately measure tumor size due to the effect of such treatment. The remaining 847 patients were reviewed in this study (Fig. 1). In each patient, pathological TNM classification was performed according to the TNM classification of malignant tumors published by the UICC (7th edition). The longest dimension measured by histopathological examination was defined as the tumor size. Tumor resectability was classified according to the National Comprehensive Cancer Network (NCCN) guideline [16]. Overall survival was defined as the interval from the date of surgery to the last follow-up date or death. Statistical analyses were performed using EZR, which is a graphical user interface for R version 2.13.0 (R Foundation for Statistical Computing, Vienna, Austria) [17], and differences were considered significant at *P* < 0.05. The ethics review board of each participating hospital approved this study.

### Calculation of tumor size cutoff values (Tco values)

Tumor size data for 90 patients (48 with T1 disease and 42 with T2 disease) were analyzed to determine the optimum Tco value for tumors limited to the pancreas. Receiver operating characteristics (ROC) analysis was performed by drawing ROC curves with tumor size as an independent variable and the 3-year survival as the outcome. In addition, the tumor size data for 676 patients with T3 disease except M1 patients were analyzed to determine the optimum Tco value for tumors extending beyond the pancreas, by drawing ROC curves with tumor size as an independent variable and the 1-, 2-, and 3-year survival as outcomes. The optimum cutoff point was defined as that with the highest sensitivity plus specificity. Two patients with T4 disease were excluded from this analysis of Tco values for tumors extending beyond the pancreas.

### Proposed new TNM classification based on Tco values

To define the new T category, tumors limited to the pancreas were reclassified as T1 or T2 using our new Tco value for these tumors, while tumors extending beyond the pancreas (T3 in the current TNM classification) were reclassified as T3a or T3b using our Tco value for such tumors. Consequently, in our proposed new TNM classification, stages IA and IB remain as T1N0M0 and T2N0M0, respectively. On the other hand, the current stage IIA (T3N0M0) becomes stage IIA (T3aN0M0) and stage IIB (T3bN0M0), and the stage IIB (T1-3N1M0) changes to stage IIC (T1-3bN1M0).

### Evaluation of the new TNM classification

New Tco values were validated separately for tumors limited to the pancreas (*n* = 90) and tumors extending beyond the pancreas (*n* = 755). Overall survival was compared between two cohorts divided at these new Tco values by univariate analysis with the log-rank test, and it was also compared between two cohorts categorized according to lymph node metastasis (N category of the TNM classification) or distant metastasis (M category of the TNM classification). The hazard ratio and its 95% confidence intervals were estimated using univariate and multivariate Cox’s proportional hazards models.

After reclassifying the 847 patients according to our new TNM classification, clinicopathological factors (age, gender, tumor site, tumor resectability, operating time, intraoperative blood loss, preoperative serum CA19-9 level, surgical margin, tumor histology, and postoperative adjuvant chemotherapy) were compared between stage IA (*n* = 36) and stage IB (*n* = 22) with a *t* test or Fisher’s exact test and were also compared among stages IIA (*n* = 103), IIB (*n* = 85), and IIC (*n* = 488) using an analysis of variance or Fisher’s exact test. To evaluate the usefulness of our modifications, survival curves based on the new or current TNM classification were constructed with the Kaplan–Meier method and compared using the log-rank test for each two-group comparison. In addition, the performance of the new and current classifications was compared using Akaike’s information criterion (AIC) [18], which is a criterion for selecting a suitable model. It is often used to compare the goodness of fit between the built models. The model with the smaller AIC value is deemed a better fit.

## Results

### Tumor size cutoff values (Tco values)

**Fig. 2:**
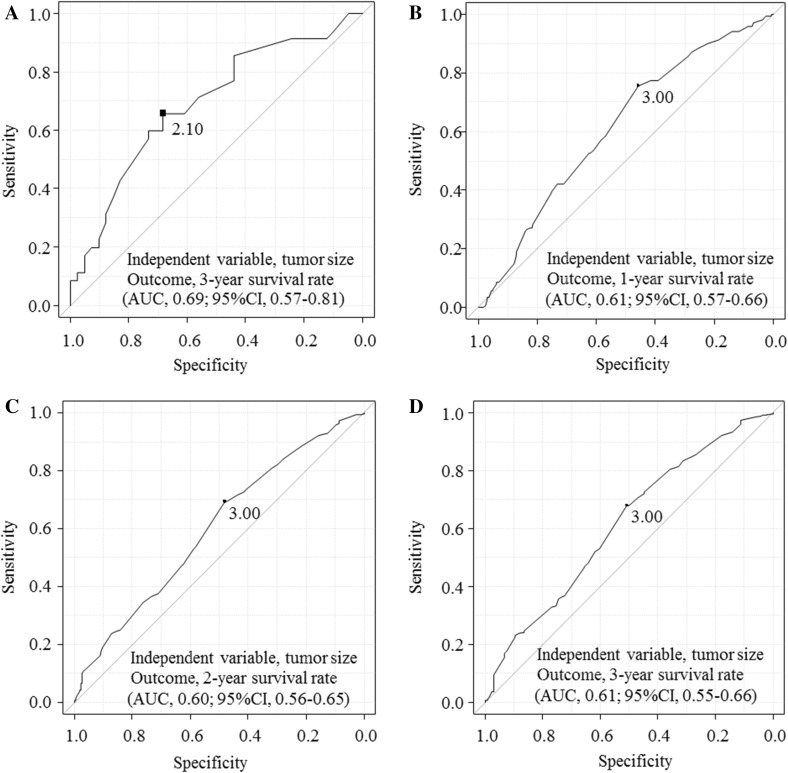
**a** Calculation of the cutoff value for tumor size (Tco value) in patients with tumors limited to the pancreas by receiver operating characteristics (ROC) analysis using tumor size as an independent variable and the 3-year survival as the outcome. The optimum cutoff point was defined as that with the highest sensitivity plus specificity. Tumor size data for 90 patients (48 with T1 and 42 with T2 disease) were analyzed, and the Tco value was calculated to be 2.1 cm. As a result, the Tco value dividing T1 and T2 in the new TNM classification was set at 2 cm. **b**–**d** Calculation of the cutoff Tco value in patients with tumors extending beyond the pancreas by ROC analysis using tumor size as an independent variable and the 1-, 2-, and 3-year survival as outcomes. Tumor size data for 755 patients with T3 disease were analyzed, and the Tco values were calculated as 3.0, 3.0, and 3.0 cm, respectively. Accordingly, the Tco value dividing T3a and T3b in the new TNM classification was set as 3 cm

The optimum Tco value for tumors limited to the pancreas was calculated to be 2.1 cm based on the ROC curve for 3-year survival, and the area under the curve (AUC) was 0.69 (95% confidence interval, 0.57–0.81; Fig. 2a). Therefore, the Tco value dividing T1 from T2 in our new TNM classification was set as 2 cm, which was identical to that in the current TNM classification. The optimum Tco values for tumors extending beyond the pancreas were calculated from the ROC curves for 1-, 2-, and 3-year survival as 3.0, 3.0, and 3.0, respectively (Fig. 2b–d). The AUC values were 0.61 (95% confidence interval, 0.57–0.66), 0.60 (0.56–0.65), and 0.61 (0.55–0.66), respectively. Accordingly, the Tco value dividing T3a from T3b in the new TNM classification was set as 3 cm.

### Usefulness of the new TNM classification

Regarding tumors limited to the pancreas, univariate analysis revealed a significant difference in overall survival between two cohorts divided at a Tco value of 2 cm (T1 and T2) as well as between two cohorts separated by N category status. Multivariate analysis indicated that the Tco value of 2 cm (hazard ratio, 2.45; 95% confidence interval, 1.32–4.56; *P* = 0.005) and the N category (hazard ratio, 1.83; 95% confidence interval, 1.01–3.32; *P* = 0.048) were both independent prognostic factors for tumors limited to the pancreas, with a Tco value of 2 cm being a more important prognosticator than the N category (Table 1). Regarding tumors extending beyond the pancreas, univariate analysis revealed significant differences of overall survival between two cohorts divided at a Tco value of 3 cm (T3a and T3b), between two cohorts separated by N category status, and between two cohorts divided by M category status. Multivariate analysis indicated that the Tco value of 3 cm (hazard ratio, 1.46; 95% confidence interval, 1.20–1.77; *P* < 0.001) and N category (hazard ratio, 1.72; 95% confidence interval, 1.37–2.16; *P* < 0.001) were both independent prognostic factors for tumors extending beyond the pancreas, although the N category was more important than the Tco value (Table 2).

**Table 1:** Univariate and multivariate survival analyses based on a tumor size of 2 cm, N category status, and M category status for patients with tumors limited to the pancreas (T1 or T2) (*n* = 90)

Variables	No. of patients (%)	MST (months)	Univariate analysis		Multivariate analysis	
			*P* value	Hazard ratio (95% CI)	*P* value	Hazard ratio (95% CI)
Tumor size (cm)			0.002	2.63 (1.41–4.91)	0.005	2.45 (1.32–4.56)
≤2	49 (54.4)	86.3				
>2	41 (45.6)	33.5				
N category			0.024	1.97 (1.08–3.54)	0.048	1.83 (1.01–3.32)
No	59 (65.6)	70.2				
Yes	31 (34.4)	33.5				
M category			0.553	0.55 (0.08–4.03)	–	–
No	87 (96.7)	55.1				
Yes	3 (3.3)	35.2				

*MST* median survival time, *CI* confidence interval

**Table 2:** Univariate and multivariate survival analyses based on a tumor size of 3 cm, N category status, and M category status for patients with tumors extending beyond the pancreas (T3) (*n* = 755)

Variables	No. of patients (%)	MST (months)	Univariate analysis		Multivariate analysis	
			*P* value	Hazard ratio (95%CI)	*P* value	Hazard ratio (95%CI)
Tumor size (cm)			<0.001	1.68 (1.39–2.03)	<0.001	1.46 (1.20–1.77)
<3	274 (36.3)	30.3				
≥3	481 (63.7)	17.5				
N category			<0.001	1.97 (1.58–2.45)	<0.001	1.72 (1.37–2.16)
No	192 (25.4)	35.7				
Yes	563 (74.6)	18.4				
M category			<0.001	1.53 (1.22–1.93)	0.077	1.24 (0.98–1.56)
No	647 (85.7)	21.9				
Yes	108 (14.3)	16.2				

*MST* median survival time, *CI* confidence interval

After reclassifying the 847 patients according to our new TNM classification, the number of patients in stages IA and IB remained at 36 and 22, respectively, while the number of patients in our new stages IIA, IIB, and IIC was 103, 85, and 488, respectively. Comparison of clinicopathological factors between stages IA and IB failed to identify any significant factors that could support the validity of this classification (Table 3). Comparison of clinicopathological factors among the new stages IIA, IIB, and IIC revealed that there were significantly more borderline resectable patients (*P* = 0.003), significantly larger intraoperative blood loss (*P* = 0.01), and a significantly higher preoperative serum level of CA19-9 in new stage IIB than new stage IIA, while the frequency of distal pancreatic tumors was significantly higher in new stage IIC than new stage IIB (Table 4).

**Table 3:** Comparison of clinicopathological characteristics between stage IA and stage IB

Parameter	Stage IA (T1N0M0) (*n* = 36)	Stage IB (T2N0M0) (*n* = 22)	Total (*n* = 58)	*P* value
Age (years)	68.6 ± 8.5	66.5 ± 7.7	67.8 ± 8.2	0.358
Gender				
Male	18 (50.0)	14 (63.6)	32	0.75
Female	18 (50.0)	8 (36.4)	26	
Tumor location				
Proximal	23 (63.9)	10 (45.5)	33	0.27
Distal	13 (36.1)	11 (50.0)	24	
Other	0 (0)	1 (0.5)	1	
NCCN resectability				
R	33 (91.7)	21 (95.5)	54	1
BR	3 (8.3)	1 (4.5)	4	
Operating time (min)	401.3 ± 131.6	391.2 ± 140.8	397.5 ± 133.9	0.79
Intraoperative blood loss (mL)	880.4 ± 626.9	1097.9 ± 777.4	961.9 ± 688.5	0.26
Serum level of CA19-9 (U/mL)	127.7 ± 345.5	199.9 ± 258.9	155.3 ± 314.7	0.41
Surgical margin				
Positive	1 (2.7)	4 (18.2)	5	0.06
Negative	35 (97.3)	18 (81.8)	53	
Tumor histology				1
Papillary	2 (5.6)	2 (9.1)	4	
Well-differentiated	11 (30.6)	6 (27.3)	17	
Moderately differentiated	23 (63.9)	10 (45.5)	33	
Poorly differentiated	0 (0)	4 (18.2)	4	
Adenosquamous	0 (0)	0 (0)	0	
Adjuvant chemotherapy				
Yes	26 (72.2)	17 (77.3)	43	0.76
No	10 (27.8)	5 (22.7)	15	

*NCCN* National Comprehensive Cancer Network, *R* resectable, and *BR* borderline resectable

**Table 4:** Comparison of clinicopathological characteristics among patients in the proposed new stages IIA, IIB, and IIC

Parameter	New stage IIA (T3aN0M0) (*n* = 103)	New stage IIB (T3bN0M0) (*n* = 85)	New stage IIC (T1-3bN1M0) (*n* = 488)	Total (*n* = 676)	*P* value*
Age (years)	71.0 ± 8.3	68.8 ± 8.9	67.2 ± 10.2	67.9 ± 9.9	0.001
Gender					0.83
Male	56 (54.4)	50 (58.8)	277 (56.8)	383	
Female	47 (45.6)	35 (41.3)	211 (43.2)	293	
Tumor location					0.014
Proximal	70 (68.0)	47 (55.3)	355 (72.7)	472	
Distal	33 (32.0)	33 (38.8)	123 (25.2)	189	
Other	0 (0)	5 (5.9)	10 (2.0)	15	
NCCN resectability					<0.01
R	84 (81.6)	52 (61.2)	291 (59.6)	427	
BR	19 (18.4)	33 (38.8)	197 (40.4)	249	
Operating time (min)	407.6 ± 154.0	433.5 ± 195.4	448.2 ± 155.5	440.3 ± 161.2	0.066
Intraoperative blood loss (mL)	998.1 ± 737.9	1450.4 ± 1579.6	1253.6 ± 1043.3	1239.6 ± 1092.2	0.018
Serum level of CA19-9 (U/mL)	281.1 ± 525.1	545.3 ± 970.7	914.1 ± 3465.9	762.1 ± 2941.2	0.142
Surgical margin					0.054
Positive	17 (16.5)	22 (25.9)	136 (27.9)	175	
Negative	86 (83.5)	63 (74.1)	352 (72.1)	501	
Tumor histology					0.015
Papillary	3 (2.9)	1 (1.2)	6 (1.2)	10	
Well-diff	31 (30.1)	24 (28.2)	114 (23.4)	169	
Moderately diff	62 (60.2)	44 (51.8)	315 (64.5)	421	
Poorly diff	7 (6.8)	9 (10.6)	43 (8.8)	49	
Adenosquamous	0 (0)	7 (8.2)	10 (2.0)	17	
Adjuvant chemotherapy					0.255
Yes	88 (85.4)	65 (76.5)	384 (79.2)	537	
No	15 (14.9)	20 (23.5)	101 (20.8)	136	

*NCCN* National Comprehensive Cancer Network, *R* resectable, *BR* borderline resectable, and *diff* differentiated

* *P* value is calculated using analysis of variance (ANOVA) for continuous variables and Fisher’s exact test for categorical variables

**Fig. 3:**
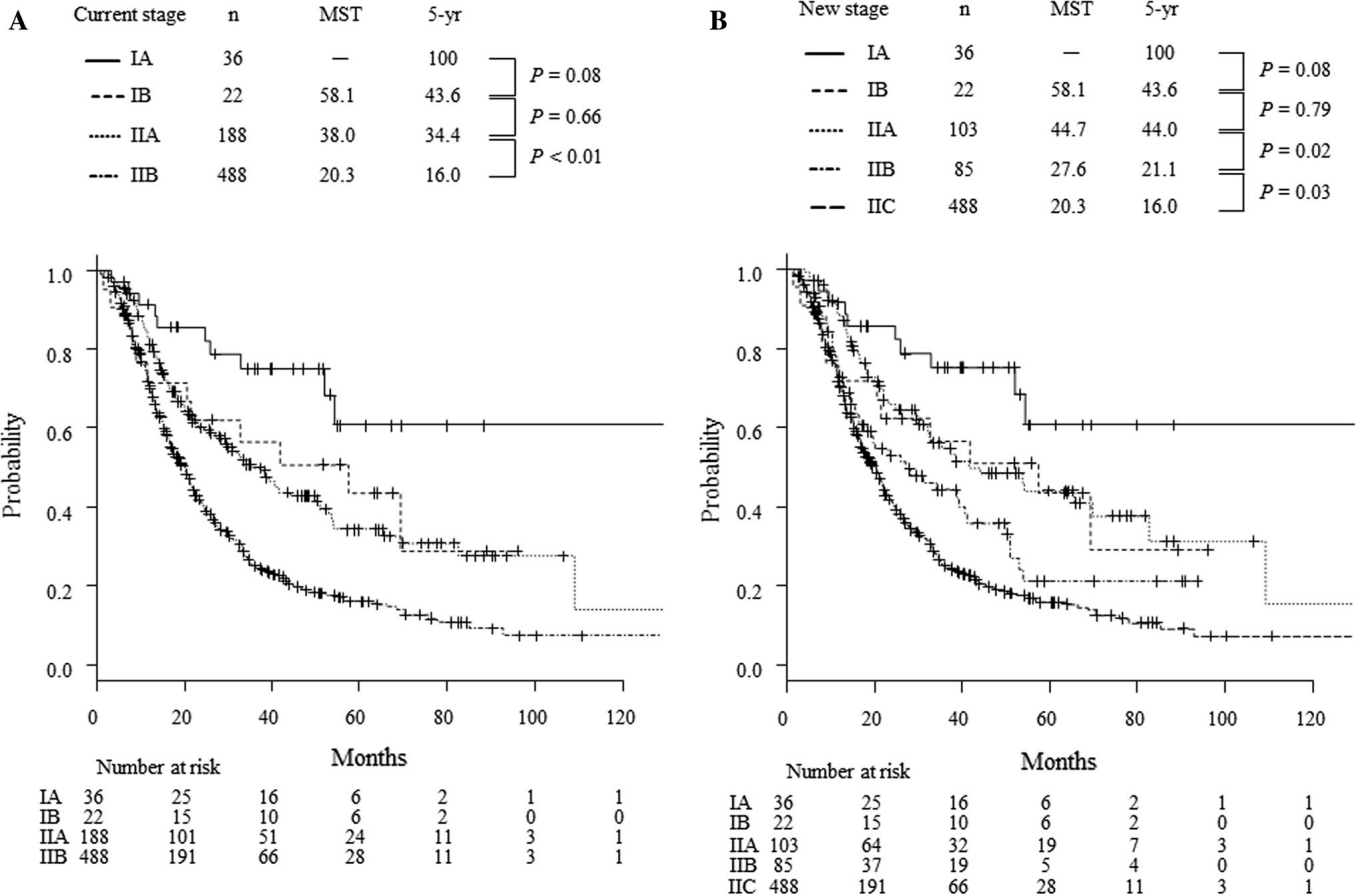
**a** Actuarial survival curves of 847 patients according to the current TNM classification. The 5-year survival rate was not significantly different between stage IA (100%) and stage IB (43.6%; *P* = 0.08) or between stage IB and stage IIA (34.4%; *P* = 0.66), while there was a significant difference between stages IIA and IIB (16.0%; *P* < 0.01). **b** Actuarial survival curves of 847 patients according to the new TNM classification. The 5-year survival rate was not significantly different between stage IB (43.6%) and the new stage IIA (44.0%; *P* = 0.79), while there were significant differences between new stages IIA and IIB (21.1%; *P* < 0.02) and between new stages IIB and IIC (16.0%; *P* = 0.03). The P value was calculated using the log-rank test for each two-group comparison

Comparison of overall survival showed no significant difference between stages IA and IB, but there was a significant difference between current stages IIA and IIB (Fig. 3a), as well as significant differences between new stages IIA and IIB and between new stages IIB and IIC (Fig. 3b).

Table 5 shows the results from the multivariate Cox’s analysis using the new and current classifications as a factor. According to the left half of Table 5, the new classification was significantly associated with overall survival, even when the prognostic factors were adjusted. In the right half of Table 5, the current classification was also significant; however, the AIC for the new classification was smaller than that for the current classification. This result indicated that the new classification outperforms the current classification.

**Table 5:** Multivariate Cox’s regression analysis for overall survival in patients in stage II

Effect and level	New stage		Current stage	
	Adjusted HR (95% CI)	*P*	Adjusted HR (95% CI)	*P*
Stage		<0.001		<0.001
2B vs 2A	1.52 (0.96,2.39)	0.072	1.94 (1.50,2.52)	<0.001
2C vs 2A	2.37 (1.67,3.38)	<0.001	–	–
Age (10 yrs)	1.36 (1.20,1.54)	<0.001	1.35 (1.19,1.54)	<0.001
Gender, M vs F	1.04 (0.82,1.32)	0.77	1.03 (0.81,1.31)	0.82
Tumor location, proximal vs distal	0.83 (0.62,1.12)	0.22	0.82 (0.62,1.10)	0.19
NCCN resectability, R vs BR	1.02 (0.79,1.31)	0.87	1.00 (0.77,1.28)	0.97
Operating time (60 min)	1.02 (0.96,1.08)	0.47	1.02 (0.96,1.08)	0.49
Intraoperative blood loss (1000 mL)	1.22 (1.08,1.37)	<0.001	1.24 (1.10,1.39)	<0.001
Serum level of CA19-9 (1000 U/mL)	1.03 (1.00,1.06)	0.047	1.03 (1.00,1.06)	0.045
Surgical margin, positive vs negative	1.12 (0.87,1.45)	0.37	1.14 (0.89,1.47)	0.30
Tumor histology		<0.001		<0.001
Well-diff vs adsq	0.22 (0.11,0.46)	<0.001	0.21 (0.10,0.43)	<0.001
Moderately diff vs adsq	0.32 (0.16,0.63)	0.001	0.29 (0.15,0.58)	<0.001
Poorly diff vs adsq	0.44 (0.21,0.94)	0.034	0.42 (0.20,0.88)	0.022
Papillary vs adsq	0.16 (0.06,0.48)	0.001	0.15 (0.05,0.43)	<0.001
Adjuvant chemotherapy, Y vs N	0.57 (0.44,0.74)	<0.001	0.57 (0.43,0.74)	<0.001
T category		0.055		0.061
2 vs 1	3.65 (1.15,11.63)	0.028	3.61 (1.13,11.48)	0.030
3 vs 1	3.39 (1.24,9.27)	0.017	3.31 (1.21,9.05)	0.019
No. of analysis data	543		543	
AIC	3674.4		3675.6	

*NCCN* National Comprehensive Cancer Network, *R* resectable, *BR* borderline resectable, *diff* differentiated, *adsq* adenosquamous, and *AIC* Akaike’s information criterion (smaller is better)

## Discussion

Many previous studies have found that tumor size was an independent prognostic factor for pancreatic adenocarcinomas, but the Tco value used to evaluate the significance of tumor size has varied among them. Based on a Tco value of 3 cm, Yeo et al. [3], Benassai et al. [8], and Winter et al. [13] reported that tumor size was an independent prognostic factor in pancreatoduodenectomy patients. Moon et al. [10] also employed 3 cm in a study that identified tumor size as an independent prognostic factor in patients who had undergone resection of pancreatic adenocarcinoma by any mode of pancreatectomy. However, Geer et al. [11] and Lim et al. [9] employed 2.5 cm and 2 cm as the Tco values, respectively, in studies of patients who underwent R0/1 pancreatectomy, while Meyer et al. [12] employed 2 cm as the Tco value to evaluate patients undergoing R0 pancreatectomy. All these studies showed that tumor size was an independent prognostic factor after resection of a pancreatic adenocarcinoma. Thus, a range from 2 to 3 cm has been employed as the Tco value in previous studies. Of these studies, only two described the stage distribution of the patients. In the study of Meyer et al. [12], 10.8% of the patients were in stages IA or IB versus only 4.3% in the study of Moon et al. [11]. In the former study, which recruited a higher percentage of patients with tumors limited to the pancreas, the Tco value was set at 2 cm, while it was 3 cm in latter study. However, the rationale for setting the Tco value was not explained in most of the previous reports. In a few studies, significant differences of survival were found by comparing different candidate Tco values set at regular intervals, and the Tco value with the lowest *P* value was selected [3, 9, 11]. However, none of the previous studies employed ROC analysis to determine the Tco value as we did this time. ROC curves can be used to statistically detect the optimum Tco value without researcher bias, which is considered to be an advantage of the present study.

In this study, multivariate analysis revealed that tumor size was the most significant independent prognostic factor for patients with tumors limited to the pancreas, but the Tco value distinguishing T1 and T2 was the same as in the current TNM classification, and there was no significant difference in overall survival between stages IA and IB. Because of the small number of patients with tumors limited to the pancreas in this study, it was difficult to assess the correlation of tumor size with their prognosis. Among patients with tumors extending beyond the pancreas, the preoperative serum level of CA19-9 was significantly higher in new stage IIB than new stage IIA. This finding corresponded to previous reports that a high preoperative serum level of CA19-9 is a significant adverse prognostic factor [19, 20]. In addition, the proportion of patients with borderline resectable tumors was significantly larger in new stage IIB than IIA, indicating that tumor size is strongly related to tumor invasion of the main arterial trunks (celiac axis and/or superior mesenteric artery) [21]. These results provide support for our proposed new TNM classification.

Many studies have found that N category status is a significant independent prognostic factor [3, 8, 9, 11, 12], although there have been a few exceptions [7, 10]. The N category status was a significant independent prognostic factor for patients who underwent R0/1 pancreatectomy in this study as well. Therefore, the current stage IIB (T1-3N1M0) was defined as the proposed new stage IIC (T1-3bN1M0), basically maintaining the composition of all three categories. The resulting new TNM classification was considered to be useful because there was a significant difference of survival between stages IIA and IIB as well as between stages IIB and IIC.

According to the NCCN guidelines of 2015, preoperative staging laparoscopy is recommended for high-risk patients (borderline resectable disease, markedly elevated CA19-9, large primary tumors, or large regional lymph nodes) [22]. Since CA19-9 > 150 U/mL and tumor size > 3 cm are considered as surrogate markers for preoperative staging laparoscopy, we insist on the necessity of performing preoperative staging laparoscopy to the patients with our new stage IIB or higher to check for unresectability prior to the surgery [23].

In conclusion, the proposed new TNM classification of pancreatic cancer developed in this study reflects tumor size, which is an important prognostic factor in patients with tumors extending beyond the pancreas (current T3), and they form the largest group undergoing pancreatectomy. The present results require validation by a large-scale study employing ROC analysis in the future.
